# Prevalence of depression and anxiety symptoms among elite female beach volleyball players in the FIVB top 200

**DOI:** 10.1007/s44192-026-00378-8

**Published:** 2026-01-24

**Authors:** Marc Niering, Niklas Engel, Rainer Beurskens, Johanna Seifert

**Affiliations:** 1 Department of Psychiatry, Social Psychiatry, and Psychotherapy, Hannover Medical School, Carl-Neuberg Straße 1, 30625 Hannover, Germany; 2 Department of Sport Psychology, Austrian Volleyball Federation, Vienna, Austria; 3 Department of Health and Social Affairs, FHM Bielefeld - University of Applied Sciences, Bielefeld, Germany; 4 Institute of Biomechanics and Neurosciences, Nordic Science, Hannover, Germany

**Keywords:** Beach volleyball, Female athletes, Depression, Anxiety, Mental health

## Abstract

**Background:**

Anxiety and depressive symptoms are common in elite sports but remain understudied in female professional beach volleyball. Unique stressors including financial insecurity, dyadic team structures, and public visibility may increase vulnerability to mental health issues.

**Objective:**

The aim of this study was to assess the prevalence and severity of anxiety and depressive symptoms among female professional beach volleyball players and to investigate potential influencing factors such as training volume, financial security, and perceived social support.

**Methods:**

This cross-sectional study included 52 professional female beach volleyball players ranked within the top 200 of the world ranking (mean age 26.14 ± 4.70 years) from German-speaking countries, the United States, and Canada. Data were collected using standardized self-report instruments, including the State Trait Anxiety Inventory Trait version (STAI-T) and the Beck Depression Inventory II (BDI-II). Additionally, psychosocial and structural stressors such as financial uncertainty, interpersonal team dynamics, and support from coaches and teammates were assessed. Descriptive statistics, correlation analyses, and multiple regression analyses were conducted to identify significant associations.

**Results:**

In total, 67.3% (n = 35) of athletes exceeded the clinical cut-off score for trait anxiety (STAI-T ≥ 44), and 71.2% (n = 37) reported depressive symptoms above the clinical threshold (BDI-II ≥ 14). Trait anxiety and depressive symptoms were strongly correlated (*r* = 0.777, *p* < 0.001). Higher training volume correlated with more severe depressive symptoms (*r* = 0.450, *p* <0 .001), and several sport-related factors showed significant correlations with trait anxiety (all *p* < 0.05). Multiple regression identified poor team communication (*p* < 0.001) and financial insecurity (*p* = 0.026) as significant predictors of psychological burden, whereas international ranking showed no association with either anxiety or depression.

**Conclusions:**

Elite female beach volleyball players exhibit high rates of clinically relevant anxiety and depressive symptoms. Psychosocial stressors, structural insecurities and subjective success perceptions, rather than objective competitive success, appear to be key contributors. These findings underscore the need for targeted sport-psychological support, financial stabilization, and long-term preventive strategies tailored to the unique demands of elite beach volleyball.

## Highlights

- 71.2% of professional female beach volleyball players reported clinically relevant symptoms of self-reported depression.
- 67.3% of athletes exceeded the clinical cut-off for trait anxiety.
- High training volume was significantly associated with more severe depressive symptoms.
- Financial insecurity and poor team communication were significant predictors of increased psychological burden.

## Introduction

Mental health problems are increasingly recognized as a major concern in elite sports, affecting not only athletic performance but also long-term well-being [49]. Among the most prevalent psychological symptoms in this population are anxiety and depression, which often remain underdiagnosed and undertreated due to stigma and performance-driven culture [18]. Recent studies indicate that elite athletes are at comparable or even elevated risk of mental health disorders compared to the general population [22].

Female athletes appear to be particularly vulnerable. A growing body of evidence shows that women in competitive sports report significantly higher rates of depression and anxiety than their male counterparts [27, 30]. Contributing factors may include hormonal variability, sociocultural pressures, financial insecurity, and a higher tendency toward perfectionism and neuroticism [6, 45]. Global data suggest that approximately 6 percent of adult women suffer from depression compared to 4 percent of men [14, 63], a pattern also observed in European cohorts [3]. Åkesdotter et al. [1] found that up to 20 percent of female elite athletes report clinically relevant symptoms of depression or anxiety at any given time, particularly in sports that require high individual responsibility and public exposure.

Within this context, volleyball is a globally practiced team sport that has gained popularity in both amateur and elite settings. Despite its widespread presence, there is a scarcity of empirical research on the mental health of active volleyball players. Studies by Vaccaro et al. [57] and Patsiaouras et al. [46] indicate that amateur players exhibit higher levels of psychological distress compared to elite athletes, but also point out that female players display lower emotional stability, which may increase their vulnerability to stress during competition.

Beach volleyball, a variant of the sport played in a two-player team format, presents a unique set of psychological and structural challenges. The high interpersonal dependency within the dyadic team structure, constant international travel, public visibility, and physical demands distinguish beach volleyball from other team sports [24, 38]. Additional pressures stem from financial instability, as many professional beach volleyball players rely on short-term sponsorships, prize money, and public funding. Previous research has linked financial insecurity and limited team communication to higher levels of anxiety and depression in athletes [18, 48].

Despite these sport-specific stressors, little is known about the mental health of professional female beach volleyball players. Existing studies have predominantly focused on indoor volleyball or mixed-gender samples, leaving a gap in understanding the unique mental health risks in this subgroup. As part of these broader psychosocial and structural stressors, elevated training loads, financial insecurity and reduced social support have each been identified as key risk factors for anxiety and depression in elite athletes [22, 36].

The present study aims to address this gap by systematically examining the prevalence and severity of anxiety and depressive symptoms in professional female beach volleyball athletes. Moreover, the study explores the role of training load, financial security, and psychosocial support as potential contributing factors. The findings are intended to inform the development of targeted preventive and therapeutic strategies tailored to the needs of this high-risk population.

## Material and methods

### Study design

This study was conducted as a cross-sectional investigation using an online survey. Data collection took place between 14th February and 18th April 2025. Due to the specific inclusion criterion that only professional elite female beach volleyball players were eligible, recruitment was carried out by directly contacting the responsible national coaches and coordinators of the Austrian Volleyball Association, the German Volleyball Association, Swiss Volley, Volleyball Canada, and USA Volleyball. All eligible athletes aged 18 years and older were invited to participate upon confirmation of their active status on the international beach volleyball circuit.

### Participants

A total of 52 professional female beach volleyball players were included in the study, all of whom met the predefined inclusion criteria regarding age, competitive level, and training volume. The athletes were recruited from German-speaking countries, the United States, and Canada. The mean age of participants was 26.14 years (SD = 4.70), with an age range from 18 to 37 years.

Eligibility criteria included a minimum age of 18 years, an official world ranking within the top 200 of the Fédération Internationale de Volleyball (FIVB) at the time of data collection, and a weekly training volume of at least 10 h, as proposed by Lima et al. (2022) to define professional-level training. In this sample, all athletes met these criteria, reporting an average training volume of 23.44 h per week (SD = 5.68) and a mean self-rated sporting success of 3.73 (SD = 0.95) on a 5-point Likert scale (1 = not successful, 5 = very successful).

To determine the required sample size, an a priori power analysis was conducted using G*Power. A one-tailed t-test for point-biserial correlations was calculated, assuming a medium effect size *d* = 0.3, a significance level (α) of 0.05, and a statistical power of 0.80. This medium effect size aligns with empirical norms in psychological research, where correlations in the range of r = 0.20 to 0.30 are most common [21]. Because our primary analyses focused on associations between dichotomous and continuous variables, a correlation-based power calculation was appropriate [15]. Based on these parameters and in accordance with established statistical standards [10], the required sample size was 64 participants. Taking into account the estimated total population of approximately 400 female beach volleyball players ranked within the top 200 of the FIVB worldwide, the sample size was adjusted to 55 using finite population correction [17]. The final sample of 52 athletes fell slightly short of the adjusted target size of 55, resulting in a marginal reduction in power. Given the high proportion of the accessible elite population represented in the dataset, the sample remains adequate for exploratory analyses, although results should be interpreted with appropriate caution. Sociodemographic characteristics are shown in Table 1.

**Table 1:** Sociodemographic characteristics of the beach volleyballers surveyed (n = 52)

Variable	n	% of participants
FIVB Ranking		
Between position 200 and 150	13	19.4
Between position 149 and 100	15	22.4
Between position 99 and 50	9	13.4
Between position 49 and 25	10	14.9
Between position 24 and 1	5	7.5
Age		
18–22 years	13	19.4
23–27 years	23	34.3
28–32 years	12	17.9
33–37 years	5	7.5

*n* number of

Participation in the study was voluntary and required informed consent after participants had been fully briefed on the study’s aims, anonymity, and data protection measures.

### Data collection

SoSci Survey (www.soscisurvey.de) was used as the survey instrument for data collection. The questionnaire was made available in two language versions (German and English). Participants received detailed information about the aims and procedure of the study before participation. Participation was voluntary and anonymous.

Symptoms of anxiety were assessed using the STAI-T, consisting of 20 items. The German version uses an eight-point Likert scale, which was recoded into four categories to allow comparability with the English version. A cut-off score of 44 was used to indicate clinically relevant anxiety symptoms [13, 28]. Internal consistency of the STAI-T in the present sample was acceptable (Cronbach’s α = 0.80; McDonald’s ω = 0.85).

Symptoms of depression were assessed using the BDI-II, a 21-item questionnaire with a four-point response scale ranging from 0 to 3. Total scores were classified as follows: 0 to 8 (no depression), 9 to 13 (minimal, not clinically relevant depression), 14 to 19 (mild depression), 20 to 28 (moderate depression), and 29 to 63 (severe depression) [53]. The BDI-II showed acceptable internal consistency in this sample (Cronbach’s α = 0.73; McDonald’s ω = 0.79).

The BDI-II and the STAI-T have both been utilized in online studies to assess the prevalence and severity of depressive and anxiety symptoms in athletic populations, such as ultra-distance runners and football professionals [42, 51]. As self-report instruments, neither provides a formal clinical diagnosis. Therefore, the present study refers to “self-reported depression” and “self-reported anxiety” based on established cut-off scores.

In addition to psychometric instruments, the questionnaire included custom items assessing psychosocial factors such as financial insecurity, perceived support from teammates and coaches, and quality of team communication. Weekly training load was assessed through self-reported weekly training hours, a method shown to be reliable in elite sport settings [8]. Financial insecurity and perceived social support were each measured with single five-point Likert items, consistent with recommended brief psychosocial screening approaches in elite athlete mental-health assessments [50].

### Statistical analysis

Correlations were used to explore bivariate associations, linear regression to examine predictors of symptom severity, and logistic regression to estimate the likelihood of clinically relevant symptom levels. Statistical analysis was conducted using Jamovi (Version 2.6.26.0). Data were first screened for normal distribution and missing values, which led to the exclusion of three datasets due to incomplete responses. Internal consistency of the STAI-T and BDI-II was evaluated using Cronbach’s α and McDonald’s ω. Descriptive statistics, including mean (M), frequencies, and standard deviation (SD), were calculated to summarize the sample characteristics and core variables.

To examine potential risk factors for the occurrence and severity of self-reported depression and anxiety, inferential statistical analyses were performed. Pearson correlation coefficients (*r*) were calculated to determine associations between the severity of depression and anxiety symptoms and sport-specific variables such as weekly training volume, self-rated success, and FIVB ranking.

To identify predictors of the presence of depression and anxiety, multivariate logistic regression analyses were conducted. For these analyses, binary outcome variables were created: BDI-II scores of 0–13 were coded as 0 (no self-reported depression), and scores of 14–63 as 1 (self-reported depression). For anxiety, a STAI-T score of ≥ 44 was defined as the threshold for clinically relevant self-reported anxiety and used to code binary variables accordingly.

In a subsequent step, multiple linear regression analyses were used to assess predictors of symptom severity for both depression and anxiety. Only variables considered relevant to competitive athletes (e.g., previous injury, training volume, FIVB ranking), as well as age, use of psychotropic medication, and current psychological treatment, were included. Variables unrelated to athletic performance (e.g., history of suicide attempts) were excluded. Model fit for logistic regressions was assessed using Nagelkerke’s pseudo-*R*^*2*^ [54]. The significance level was set at *p* ≤ 0.05 for all statistical tests [7].

## Results

### Prevalence and severity of self-reported symptoms of depression and anxiety

Overall, 21.15% (n = 11) of the participants showed no signs of self-reported depression, while 7.69% (n = 4) reported minimal depressive symptoms, which are not considered clinically relevant. A total of 71.15% (n = 37) of the participants had clinically relevant levels of self-reported depressive symptoms (i.e., BDI-II score ≥ 14). Among these athletes (n = 37), the average BDI-II score was 19.86 ± 4.49, corresponding to moderately severe symptoms. Regarding severity, 36.54% (n = 18) had mild, 28.85% (n = 15) had moderate, and 5.77% (n = 3) had severe depressive symptoms (Tables 2, 3).

**Table 2:** Prevalence and severity of self-reported depressive symptoms

BDI-II severity of depressive symptoms (BDI-II cut-off scores)	Participants (n = 52)	BDI-II score mean (SD)	% of participants (n = 52)
No (0–8)	11	4.91 (2.47)	21.15
Minimal (not clinically relevant; (9–13)	4	11.25 (1.71)	7.69
Clinically relevant depression (14–63)	37	19.86 (4.49)	71.15
Mild (14–19)	18	16.39 (1.46)	36.54
Moderate (20–28)	15	22.23 (1.98)	28.85
Severe (29–63)	3	30.0 (1.73)	5.77

*n* number of, *BDI-II* Beck Depression Inventory Revised, *SD* standard deviation

**Table 3:** Prevalence and severity of self-reported anxiety symptoms

STAI-T severity of anxiety symptoms (STAI-T cut-off scores)	Participants (n = 52)	STAI-T score mean (SD)	% of participants (n = 52)
Not clinically relevant (< 44)	15	35.27 (5.3)	28.85
Clinically relevant anxiety (≥ 44)	37	50.92 (4.13)	71.15

*n* number of, *STAI-T* Stait Trait Anxiety Inventory-trait version, *SD* standard deviation

In terms of self-reported anxiety, 28.85% (n = 15) of the participants scored below the clinical threshold, while 71.15% (n = 37) exceeded the cut-off score of 44 on the STAI-T, indicating clinically relevant trait anxiety. Among those athletes with elevated anxiety (n = 37), the average STAI-T score was 50.92 ± 4.13.

### Group differences in BDI-II and STAI-T scores

Table 4 shows differences in BDI-II and STAI-T scores related to psychological variables and injury status in professional female beach volleyball players. Athletes with a previous diagnosis of depression reported higher BDI-II scores (*M* = 19.0) than those without such a diagnosis (*M* = 15.7), although the difference was not statistically significant (*t*(50.0) = − 1.04, *p* = 0.302). Similarly, athletes currently using psychotropic medication had higher BDI-II scores (*M* = 18.8) compared to those not using such medication (*M* = 15.4), but this difference was also not statistically significant (*t*(50.0) = − 1.32, *p* = 0.192).

**Table 4:** Depressive and anxiety symptom differences by clinical, treatment-related, and injury variables among beach volleyball players

Variable	Cat	n	BDI-II M	*t* Test	STAI-T M	*t* Test
Past diagnosis of depression	Yes	6	19.0	*t*(50.0) = − 1.04, *p* = *0.302*	47.8	*t*(50.0) = − 0.438, *p* = *0.663*
	No	46	15.7		46.2	
Past diagnosis of anxiety disorder	Yes	2	12.5	*t*(50.0) = 0.686, *p* = *0.496*	46.0	*t*(50.0) = 0.0684, *p* = *0.946*
	No	50	16.2		46.4	
Current use of psychotropic drugs	Yes	10	18.8	*t*(50.0) = − 1.32, *p* = *0.192*	50.8	*t*(50.0) = − 1.88, *p* = *0.066*
	No	42	15.4		47.0	
In psychological treatment	Yes	11	13.7	*t*(50.0) = − 1.17, *p* = *0.247*	45.4	*t*(50.0) = − 0.457, *p* = *0.649*
	No	41	16.7		46.7	
Wants psychological treatment	Yes	25	18.0	*t*(50.0) = − 1.84, *p* = *0.071*	49.3	*t*(50.0) = − 2.49, *p* = *0.016*
	No	27	14.3		43.7	
Serious injury/illness in the last 6 months	Yes	11	20.7	*t*(50.0) = − 2.48, *p* = *0.016*	50.5	*t*(50.0) = − 1.84, *p* = *0.072*
	No	41	14.8		45.3	

*t* t-value, *p* probability value; *p* < 0.05 indicates statistical significance

Participants who reported a desire to receive psychological treatment showed notably higher STAI-T scores (*M* = 49.3) than those who did not (*M* = 43.7), and this difference was statistically significant (*t*(50.0) = − 2.49, *p* = 0.016). A similar, though not statistically significant, pattern was observed for those currently in psychological treatment (*STAI-T*: *M* = 45.4 vs. 46.7; *t*(50.0) = − 0.457, *p* = 0.649) and those with a history of anxiety diagnosis (*STAI-T*: *M* = 46.0 vs. 46.4; *t*(50.0) = 0.684, *p* = 0.946).

Additionally, athletes who experienced a serious injury or illness within the past six months showed significantly elevated BDI-II scores (*M* = 20.7) compared to those without such experiences (*M* = 14.8; *t*(50.0) = − 2.48, *p* = 0.016). STAI-T scores were also higher in this group (*M* = 50.5 vs. 45.3), but the difference missed statistical significance (*t*(50.0) = − 1.84, *p* = 0.072).

*Cat* category, *n* number of participants, *BDI-II* Beck Depression Inventory–II, *STAI-T* State Trait Anxiety Inventory-trait version, *M* mean, *t* t-value, *p* probability value

### Correlation analysis of beachvolleyball specific variables with the BDI-II and STAI-T scores

The results of the correlation analysis for beach volleyball-specific variables are shown in Table 5. Regarding depressive symptoms, a statistically significant moderate positive correlation was found with weekly training volume (*r* = 0.450, *p* < 0.001), indicating that higher training loads were associated with more severe depressive symptoms. Self-reported success in sport also correlated positively with BDI-II scores (*r* = 0.425, *p* = 0.002), as did team communication (*r* = 0.401, *p* = 0.003). In contrast, age (*r* = 0.097, *p* = 0.492), FIVB ranking (*r* = − 0.004, *p* = 0.977), perceived financial support (*r* = − 0.184, *p* = 0.192), financial stress (*r* = 0.202, *p* = 0.151), support from teammates (*r* = 0.169, *p* = 0.231), and psychological influence of coaching staff (*r* = 0.268, *p* = 0.055) were not significantly correlated with BDI-II scores.

**Table 5:** Correlation of training volume, self-assessed success in sport, training experience, age, and psychosocial factors with BDI-II and STAI-T scores

Variable (reference)	Correlation *r* with the BDI-II score	*p*-value	Correlation *r* with the STAI-T score	*p*-value
Age in years	0.097	0.492	0.034	0.810
Training volume in h/week	0.450	< 0.001*	0.316	0.023
FIVB ranking	− 0.004	0.977	0.013	0.929
Self-reported success in sport	0.425	0.002	0.366	0.008
Financial security	0.023	0.874	0.076	0.591
Perceived financial support	− 0.184	0.192	− 0.313	0.024
Perceived financial stress	0.202	0.151	0.117	0.408
Support from teammate	0.169	0.231	0.037	0.797
Team communication	0.401	0.003	0.377	0.006
Psychological impact of coaching/support staff	0.268	0.055	0.185	0.189

*BDI-II* Beck Depression Inventory–II, *STAI-T* State Trait Anxiety Inventory-trait version, *r* Pearson correlation coefficient, *p* probability value; *p* < 0.05 indicates statistical significance

For anxiety symptoms, several variables showed statistically significant correlations, including training volume, self-reported success, perceived financial support and team communication (all *p* < 0.05). The remaining variables did not demonstrate significant associations.

### Logistic regression analysis to determine beachvolleyball specific predictors of the occurrence of self-reported depression and anxiety

The results of the multivariate logistic regression analyses for the occurrence of self-reported depression and anxiety are presented in Tables 6 and 7, respectively.

**Table 6:** Results of the logistic regression analysis of self-reported depression

Variable (reference)	Odds Ratio	*z*-value	degrees of freedom	*p*-value
Age in years	0.21	− 2.17	1	0.03
Training volume in h/week	0.01	0.06	1	0.95
FIVB ranking	1.08	1.32	1	0.19
Self-reported success in sport	2.11	1.65	1	0.10
Financial security	0.33	0.20	1	0.84
Perceived financial support	− 3.96	− 1.72	1	0.09
Perceived financial stress	− 0.41	− 0.45	1	0.65
Support from teammate	1.11	1.12	1	0.25
Team communication	2.12	1.91	1	0.06
Psychological impact of coaching/support staff	1.98	1.73	1	0.09

*OR* odds ratio, *z* z-value, *df* degrees of freedom, *p* probability value; *p* < 0.05 indicates statistical significance

**Table 7:** Results of the logistic regression analysis of self-reported anxiety

Variable (reference)	Odds ratio	*z*-value	degrees of freedom	*p*-value
Age in years	0.17	1.22	1	0.22
Training volume in h/week	0.12	1.07	1	0.29
FIVB ranking	1.07	1.72	1	0.09
Self-reported success in sport	− 0.36	0.47	1	0.64
Financial security	− 0.15	− 0.88	1	0.93
Perceived financial support	− 2.61	− 1.47	1	0.15
Perceived financial stress	− 0.43	− 0.73	1	0.47
Support from teammate	0.21	0.40	1	0.69
Team communication	2.06	2.25	1	0.03
Psychological impact of coaching/support staff	0.57	0.92	1	0.36

*OR* odds ratio, *z* z-value, *df* degrees of freedom, *p* probability value; *p* < 0.05 indicates statistical significance

For depression (Table 6), the regression model was statistically significant compared to a null model (*χ*^*2*^(256) = value not reported, *p* < 0.001). The model indicated that higher age (*OR* 0.21, *p* = 0.03) was significantly associated with lower odds of self-reported depression. Other variables, such as training volume (*OR* 0.01, *p* = 0.95) and FIVB ranking (*OR* 1.08, *p* = 0.19), were not significantly associated with depression.

Additionally, perceived financial security (*OR* 0.33, *p* = 0.84), perceived financial stress (*OR* − 0.41, *p* = 0.65), team communication (*OR* 2.12, *p* = 0.06), and psychological influence of coaching staff (*OR* 1.98, *p* = 0.09) did not reach statistical significance. No significant predictive value was found for support from teammates (*OR* 1.11, *p* = 0.25) or self-reported sporting success (*OR* 2.11, *p* = 0.10).

Regarding anxiety (Table 7), only team communication reached statistical significance (*OR* 2.06, *p* = 0.03), suggesting that poor communication within the team may increase the likelihood of clinically relevant anxiety symptoms.

All other variables in the model, including age (*OR* 0.17, *p* = 0.22), training volume (*OR* 0.12, *p* = 0.29), FIVB ranking (*OR* 1.07, *p* = 0.09), and perceived financial support (*OR* − 2.61, *p* = 0.15), did not significantly predict anxiety. Likewise, no statistically significant associations were observed for self-reported success in sport (*OR* 0.36, *p* = 0.64), financial stress (*OR* − 0.43, *p* = 0.47), support from teammates (*OR* 0.21, *p* = 0.69), or psychological influence of coaching staff (*OR* 0.57, *p* = 0.36).

### Linear regression analysis to determine beachvolleyball specific predictors of the BDI-II and STAI-T scores

The results of the linear regression analysis for predictors of self-reported depressive symptoms are presented in Table 8. The model was statistically significant overall (*F*(8, 256) = value not reported, *p* < 0.001), with a corrected coefficient of determination of *R*^*2*^ = 0.15. Weekly training volume emerged as a marginally significant positive predictor of depressive symptom severity (*β* = 0.37, *p* = 0.05), indicating that higher training hours were associated with more pronounced depressive symptoms.

**Table 8:** Results of the linear regression of self-reported depression

Variable (reference)	β*	*t*-value	*p*-value
Age in years	0.22	1.00	0.32
Training volume in h/week	0.37	2.01	0.05
FIVB ranking	0.58	0.75	0.46
Self-reported success in sport	1.94	1.56	0.12
Financial security	− 6.83	− 2.50	0.02
Perceived financial support	− 2.87	− 1.23	0.23
Perceived financial stress	− 0.11	− 0.14	0.89
Support from teammate	0.96	1.12	0.27
Team communication	1.47	1.57	0.13
Psychological impact of coaching/support staff	1.72	1.80	0.08

*β* standardized regression coefficient, *t* t-value, *p* probability value; *p* < 0.05 indicates statistical significance

Additionally, financial security showed a significant negative association with depressive symptoms (*β* = − 6.83, *p* = 0.02), suggesting that athletes who perceived themselves as financially secure reported fewer depressive complaints. All other variables—including age (*β* = 0.22, *p* = 0.32), FIVB ranking (*β* = 0.58, *p* = 0.46), self-reported sporting success (*β* = 1.94, *p* = 0.12), team communication (*β* = 1.47, *p* = 0.13), and psychological impact of coaching staff (*β* = 1.72, *p* = 0.08)—did not reach statistical significance.

Table 9 displays the results of the linear regression model predicting the severity of self-reported anxiety symptoms. The overall model did not yield significant predictors at the *p* < 0.05 level, although perceived financial support showed a trend-level negative association with anxiety (*β* = − 5.59, *p* = 0.05), indicating that better financial backing may contribute to lower anxiety scores.

**Table 9:** Results of the multiple linear regression analysis predicting STAI-T scores

Variable (reference)	β*	*t*-value	*p*-value
Age in years	0.30	1.18	0.25
Training volume in h/week	0.20	0.93	0.36
FIVB ranking	1.00	1.11	0.27
Self-reported success in sport	1.27	0.90	0.38
Financial security	− 4.93	− 1.57	0.16
Perceived financial support	− 5.59	− 2.08	0.05
Perceived financial stress	− 0.38	− 0.40	0.69
Support from teammate	0.39	0.40	0.69
Team communication	1.62	1.51	0.14
Psychological impact of coaching/support staff	2.01	1.83	0.08

*β* standardized regression coefficient, *t* t-value, *p* probability value; *p* < 0.05 indicates statistical significance

Other variables, including age (*β* = 0.30, *p* = 0.25), weekly training volume (*β* = 0.20, *p* = 0.36), FIVB ranking (*β* = 1.00, *p* = 0.27), self-reported success in sport (*β* = 1.27, *p* = 0.38), and team communication (*β* = 1.62, *p* = 0.14), were not significantly associated with STAI-T scores. Similarly, perceived financial stress (*β* = − 0.38, *p* = 0.69), support from teammates (*β* = 0.39, *p* = 0.69), and the psychological influence of coaching staff (*β* = 2.01, *p* = 0.08) did not reach statistical significance.

## Discussion

This study is, to our knowledge, the first to systematically assess the prevalence and correlates of anxiety and depressive symptoms in a sample of elite female beach volleyball players ranked within the FIVB top 200. The main findings were: (i) more than two-thirds of the athletes reported clinically relevant levels of both depression and trait anxiety; (ii) higher training volume was associated with more severe depressive symptoms; (iii) higher training volume, greater self-rated success, lower perceived financial support and poorer team communication were associated with more severe anxiety symptoms; (iv) poor team communication and financial insecurity emerged as significant predictors of psychological burden, and (v) no significant associations were found between international ranking and mental health outcomes. In the following sections, these findings are discussed in the context of existing literature, with particular focus on sport-specific risk factors and potential preventive strategies.

### Mental health burden: prevalence of depressive and anxiety symptoms

The present study revealed a very high prevalence of clinically relevant self-reported symptoms of depression (71.2%) and trait anxiety (67.3%) among elite female beach volleyball players ranked within the FIVB top 200. These values clearly exceed estimates from general population surveys, where prevalence rates for clinically relevant depressive symptoms typically average around 5% [32]. In elite athletes, a systematic meta-analysis reported prevalence rates of up to 34% for combined anxiety and depression, which still remains lower than the rates observed in our sample [22].

Sex-specific evidence further underscores the vulnerability of female athletes. A recent meta-analysis found that women in elite sport have a 17% higher relative risk for anxiety symptoms and a 42% higher relative risk for depressive symptoms compared to their male counterparts [33]. Consistent with this, Åkesdotter et al. [1] found that 26.0% of female elite athletes met clinical cut-offs for anxiety or depression, compared to 10.2% of male athletes. In a larger cohort, Åkesdotter et al. [2] further reported that female athletes across multiple Olympic sports scored higher on measures of depression, anxiety, and burnout than their male counterparts.

First, the present study employed comparatively sensitive cut-off values (BDI-II ≥ 14; STAI-T ≥ 44), capturing even mild symptomatology. However, the BDI-II cut-off of 14 is commonly used in clinical screening and demonstrates acceptable sensitivity and specificity in validation studies [61]. While specific validations in elite athlete samples are scarce, comparable thresholds have been applied in athletic contexts, including studies with concussed high school athletes [62], elite Para-athletes [4], and ultra-endurance runners [42]. By contrast, other research in elite sport has employed higher thresholds [35, 57] or less symptom-specific screening tools such as the GHQ-12 [16] or HADS-D [60], which are likely to yield lower prevalence estimates [11, 39].

Second, data collection occurred between February and April, a period coinciding with the build-up to and onset of the international competition season, during which training loads, travel demands, and logistical stressors are typically elevated in elite beach volleyball [38]. These conditions may be associated with increased psychological strain compared to off-season or recovery periods in which many comparable studies have been conducted. Third, the sample consisted exclusively of high-ranking, actively competing female athletes, a demographic consistently shown to have higher rates of anxiety and depression than mixed-gender or lower-competition-level cohorts [1, 33].

### Risk factor: high training volume

In the present study, higher weekly training volume was associated with more severe self-reported depressive and anxiety symptoms, supporting the hypothesis that excessive training load may be a psychological stressor in elite athletes. Similar associations have been reported in competitive adolescent athletes [23] and in Swiss elite athletes, where higher training loads were accompanied by increased depressive symptoms and greater overall psychological distress, particularly during intensified training periods [31]. Similarly, intensified training periods have been shown to elicit adverse changes in mood, increased fatigue, and reduced psychological well-being, which in most cases normalize following a reduction in load [52]. High training loads have also been linked to disturbed sleep and impaired recovery in elite athletes, factors that are strongly associated with heightened vulnerability to both depressive and anxiety symptoms [58].

Several mechanisms may explain this relationship. High training volumes have been associated with cumulative fatigue, endocrine disturbances, and sleep disruption, all of which are linked to mood dysregulation and increased vulnerability to depressive symptoms [9]. These physiological and psychological strain responses overlap substantially with mechanisms known to be associated with elevated anxiety symptoms in elite athletes, particularly under conditions of persistent high load and inadequate recovery [31]. Furthermore, sustained high loads may reduce the time available for recovery and non-sport-related activities, leading to a narrowing of social and psychological resources. While regular physical activity is generally protective for mental health [26], the present results support the growing consensus that there is a threshold beyond which training may become detrimental to psychological well-being, particularly in the absence of adequate recovery.

However, not all studies have found a positive association between training volume and depressive symptoms. For example, no significant link between training volume and depressive symptoms was found in ultra-distance runners, where younger age was a significant predictor [42], or in German elite athletes, where perceived stress and recovery balance were more relevant [43]. Evidence directly linking training volume to anxiety symptoms remains limited, suggesting that anxiety-related effects may depend strongly on contextual and sport-specific factors. Such discrepancies may also be attributable to differences in sport-specific demands, the timing of data collection within the competitive season, the psychological meaning attributed to high training loads, or individual resilience factors. These inconsistencies highlight the need for longitudinal, sport-specific investigations that can better disentangle causal relationships.

### Interpersonal dynamics: the role of team communication

Poor team communication emerged as a significant predictor of clinically relevant anxiety symptoms in this cohort, and showed a trend-level association with both depression severity and the occurrence of depressive symptoms. This finding is consistent with previous research indicating that social support and effective interpersonal communication within sporting environments act as protective factors against mental health problems [47, 59]. In elite sport, inadequate communication can contribute to interpersonal conflict, reduced psychological safety, and a heightened perception of performance pressure, factors that are strongly linked to anxiety and burnout risk [59]. This pattern aligns with the broader correlation results, where lower perceived financial support and reduced self-rated success were also significantly associated with higher anxiety symptom levels.

The dyadic team structure of beach volleyball may amplify these effects, as each athlete relies exclusively on a single partner for tactical execution, emotional regulation, and in-game decision-making. This high degree of interpersonal dependency increases the psychological impact of communication breakdowns. Evidence from collegiate sport also suggests that stronger team cohesion is associated with lower levels of self-reported anxiety and depression, mediated by reduced self-criticism and improved perceived competence [12].

### Economic stress: financial insecurity and psychological distress

Financial insecurity was identified as a significant predictor of increased depressive and anxiety symptom severity in the present study, supporting the notion that economic instability is a critical psychosocial stressor in elite sport. While the majority of research on athlete mental health has focused on performance- or injury-related factors, qualitative work with professional sportswomen has highlighted the detrimental effects of limited financial support on life satisfaction, career longevity and psychological well-being, including heightened worry and anxiety under unstable income conditions [40]. In elite beach volleyball, reliance on short-term sponsorships, fluctuating prize money, and inconsistent national funding may exacerbate uncertainty, and may be associated with sustained psychological distress.

Comparable findings have been reported in longitudinal cohorts of elite athletes, where periods of increased “financial fears” were associated with higher depression scores, independent of training load and competitive success [31]. Broader consensus work further identifies financial pressure as a key psychosocial risk factor for anxiety symptoms across elite sport [50]. The mechanism may involve chronic stress activation through persistent concerns about career stability and income security, which has been associated with reduced coping resources and exacerbate vulnerability to mood disorders.

### Performance paradox: no link to objective success indicators

In contrast to common assumptions that higher competitive success would correlate with better mental health, the present study found no significant association between international FIVB ranking and self-reported symptoms of depression or anxiety. This pattern contrasts with the correlation we observed for self-rated success, which was significantly associated with anxiety symptoms, suggesting that subjective and objective success indicators may capture different psychological processes. A similar pattern regarding objective indicators was observed by Åkesdotter et al. [2], who found no protective effect of competitive level on mental health outcomes in Olympic athletes. This aligns with previous research suggesting that objective performance metrics are often poor predictors of psychological well-being in elite athletes [37, 47]. Instead, psychosocial factors, such as perceived social support, resilience, and work-life balance, tend to exert stronger influences on mental health outcomes than competitive achievements.

One possible explanation is that success at the highest levels may introduce unique stressors, including increased media scrutiny, performance expectations, and fear of loss of status, which can offset potential mental health benefits of winning [44]. Additionally, maintaining elite performance often requires sustaining high training loads and competitive intensity, both of which may independently contribute to psychological distress [25].

However, there is also evidence suggesting that sporting success can act as a protective factor for mental health, particularly when embedded within favourable psychosocial conditions. Nezhad and Besharat [41] reported that athletes with higher competitive performance levels exhibited fewer symptoms of depression and anxiety when supported by strong coping strategies and robust social networks. Similarly, Souter et al. [55] found that in professional Australian football, older and more successful players tended to report lower anxiety scores than their less experienced counterparts, indicating that sustained success, when coupled with experience, may mitigate certain mental health risks. Further evidence from professional soccer shows that periods of sustained competitive success are associated with lower self-reported anxiety and depression, although these benefits often prove temporary and context-dependent [34].

In light of the present findings, these previously observed benefits of competitive success were not evident among elite female beach volleyball players. Several sport-specific factors may explain this discrepancy. Beach volleyball is characterised by a two-athlete team structure, continuous international travel, and a high frequency of competition within a condensed season, all of which may limit opportunities for recovery and social support [19]. Moreover, as partnerships in this sport are often short-lived and rankings can fluctuate rapidly due to the tournament format [20], competitive success may be experienced as fragile and transient, potentially reducing its protective value for mental health. These contextual demands could contribute to the absence of a measurable association between international ranking and symptoms of depression or anxiety in the current sample, despite evidence of such relationships in other elite sport settings. Taken together, these patterns highlight that the psychological relevance of success in elite sport may depend more on subjective appraisal than on objective ranking.

### Limitations

Several limitations should be considered when interpreting the findings of this study. The cross-sectional design precludes causal inferences regarding the observed associations between psychological symptoms and sport-specific variables. Longitudinal studies are required to clarify temporal relationships and potential bidirectional effects. In addition, data were collected via self-report questionnaires, which, although widely validated for use in different populations [5, 28], are susceptible to recall bias and social desirability effects. Additionally, several psychosocial variables were assessed using single-item measures, which may have reduced measurement precision despite their common use in athlete mental health research [50]. Within elite sport, stigma surrounding mental health may also lead to underreporting of symptoms, and the use of self-report screening instruments, which do not provide clinical diagnoses and show only moderate agreement with structured clinical interviews, may further affect the accuracy of case classification [29, 56]. Moreover, although the sample included athletes from multiple countries, recruitment was limited to those ranked within the FIVB top 200 and actively competing during the study period, which may reduce generalizability to lower-ranked professional players or those on career breaks. The modest sample size, though adequate for exploratory analyses, may have limited the statistical power to detect small to moderate effects in the regression models. Finally, given the sport-specific demands and structural characteristics of professional beach volleyball, caution is warranted in extrapolating these findings to other sports or mixed-gender samples.

## Conclusions

This study reveals a substantial mental health burden among elite female beach volleyball players, with more than two-thirds reporting clinically relevant symptoms of depression and anxiety. Training volume, team communication, self-rated success and financial security emerged as key correlates, while no association was observed between international ranking and psychological outcomes. These findings suggest that elite female beach volleyball players may constitute a particularly high-risk subgroup within elite sport, underscoring the urgency of implementing systematic, sport-specific screening procedures and preventive interventions.

Practical measures should include regular mental health screening, individualized training load monitoring and periodization, and the integration of psychological recovery strategies to mitigate the risk of overtraining-related mental health problems. Targeted communication and cohesion programs, such as structured communication training, conflict-resolution workshops, and coach-facilitated debriefings, may strengthen dyadic cohesion and buffer against anxiety-related performance impairment. Stabilizing financial conditions through multi-year contracts, athlete stipends, and targeted sponsorship programs, alongside financial literacy and career planning services, could help reduce the psychological burden associated with economic insecurity.

Importantly, mental health strategies should address not only underperforming athletes but also those achieving at the highest levels, as high-ranking athletes may experience hidden distress and perceive greater stigma around acknowledging mental health challenges. Embedding confidential psychological services within the training and competition environment may further reduce barriers to help-seeking.

Future research should adopt longitudinal and intervention-based designs to clarify causal pathways and test the effectiveness of preventive strategies in this population. Qualitative studies could provide valuable insights into the lived experiences of elite female beach volleyball players, informing the development of tailored, context-specific interventions. A coordinated effort involving athletes, coaches, federations, and sport psychologists is essential to safeguard and promote mental well-being in this high-performance yet high-risk sport.

## Data Availability

The datasets used and/or analysed during the current study are available from the corresponding author on reasonable request.
